# Oil-Soluble Exogenous Catalysts and Reservoir Minerals Synergistically Catalyze the Aquathermolysis of Heavy Oil

**DOI:** 10.3390/molecules28196766

**Published:** 2023-09-22

**Authors:** Yongfei Li, Shu Zhang, Ying Wang, Guobin Qi, Tao Yu, Xin Xin, Bin Zhao, Gang Chen

**Affiliations:** 1 Shaanxi University Engineering Research Center of Oil and Gas Field Chemistry, Xi’an Shiyou University, Xi’an 710065, China; yfli@xsyu.edu.cn (Y.L.); 180708@xsyu.edu.cn (T.Y.); 2 Shaanxi Province Key Laboratory of Environmental Pollution Control and Reservoir Protection Technology of Oilfields, Xi’an Shiyou University, Xi’an 710065, China; 3 Zhejiang Huayou Cobatl Company Limited, Quzhou 314500, China; 4 Technical Monitoring Center of Changqing Oilfield Company, Xi’an 710021, China; wying001_cq@petrochina.com.cn; 5 CCDC Changqing Downhole Technolegy Company, Xi’an 710018, China; qigb_cq@petrochina.com.cn; 6 Department of Crop Soil Sciences, Washington State University, Pullman, WA 99163, USA; xin.xin@wsu.edu; 7 Department of Statistics, North Dakota State University, Fargo, ND 58102, USA; bin.zhao@ndsu.edu

**Keywords:** reservoir minerals, exogenous catalysts, synergistic catalysis, heavy oil, hydrothermal cracking, model compounds

## Abstract

Oil is the “blood” and economic lifeblood of modern industry, but traditional light crude oil has been over-consumed, and it has been difficult to meet human demand for energy, so the exploitation of heavy oil is particularly important. In this paper, an oil-soluble catalyst was synthesized to catalyze the pyrolysis reaction of heavy oil in collaboration with reservoir minerals, so as to achieve efficient viscosity reduction of heavy oil and reduce production costs. The experimental results showed that Zn(II)O + K had the best synergistic viscosity reduction effect after the aquathermolysis of No. 1 oil sample under the reaction conditions of 180 °C, 4 h, 30% of water, and 0.2% of catalyst, respectively, and the viscosity reduction rate was 61.74%. Under the catalysis of the isopropanol system, the viscosity reduction rate was increased to 91.22%. A series of characterizations such as freezing point, thermogravimetric analysis, DSC analysis, component analysis, gas chromatography, wax crystal morphology analysis, and GC-MS analysis of aqueous organic matter were carried out on heavy oil after reaction by different reaction systems, and it could be verified that the viscosity of heavy oil was reduced. Finally, through the study of the reaction mechanism of the model compound, combined with the aqueous phase analysis, it can be clearly found that the depolymerization between macromolecules, the breaking of heteroatom chains, hydrogenation, ring opening, and other effects mainly occur during the reaction, thereby weakening the van der Waals force and hydrogen bond of the recombinant interval, inhibiting the formation of grid structure in heavy oil and effectively reducing the viscosity of heavy oil.

## 1. Introduction

At present, the oil fields developed earlier around the world have been overexploited, their crude oil production has declined sharply, and the oil fields have generally entered a stage of high water content. Traditional light crude oil has been overconsumed, and it has been difficult to meet human demand for energy. With economic and social development, global oil demand is expected to increase by more than 40% by 2025, according to the results of the US Geological Survey. Heavy oil resources are very rich, geological reserves of about 815 billion tons, accounting for about 70% of the world’s total oil reserves, much larger than traditional crude oil [1,2], mainly distributed in Canada, Venezuela, Russia, the United States, and China. Therefore, the extraction of heavy oil is particularly important [3]. Due to the limited supply of conventional oil and soaring oil demand, China’s oil security will face unprecedented challenges. As the world’s largest developing country, the Chinese government has made ensuring a stable oil supply an important national strategy to meet the demand for oil. China is the world’s largest crude oil importer, with the rapid development of China’s economy, of which oil imports have maintained steady growth, and China’s oil imports reached 502 million tons by 2022. Therefore, the development of unconventional crude oil has been recognized as an important and realistic option for China to offset the impact of its decline in conventional oil production and improve its oil security [4,5].

Among China’s oil and gas resources, heavy oil content accounts for about 25% of petroleum geological resources, about 20 billion tons, of which recoverable resources exceed 4 billion tons [6]. China’s heavy oil is mainly distributed in Shengli Oilfield, Liaohe Oilfield, Tahe Oilfield, Tuha Oilfield, and Zhongyuan Oilfield [7]. Due to the extremely complex nature of heavy oil, it is extremely difficult to extract heavy oil, and the complex formation structure will also add additional difficulties to the exploitation of heavy oil. Heavy and extra-heavy oils are characterized by high gelatinous and asphaltene content, which complicates the transportation and refining of oil and leads to an increase in the cost of heavy oil-refining products. In practice, “hydrothermal cracking” technology is widely used, that is, in a high temperature range, water is used as a catalyst to accelerate the breaking of chemical bonds in heavy oil molecules, so that the recombinant parts are converted into light components, and the content is reduced, resulting in a decrease in the viscosity of heavy oil [8,9].

In situ viscosity reduction of heavy oil water thermal cracking fundamentally solves the problem of the high viscosity of heavy oil, so it has created wide interest among researchers, among which the core of hydrothermal cracking technology is the catalyst. The oil-soluble catalyst can fully contact with the oil phase, which can better improve the efficiency of catalyzing the heavy oil water thermal cracking reaction and reduce the viscosity of heavy oil [10,11]. Zhao et al. [12] successfully reduced the viscosity of Liaohe heavy oil by more than 90% using nickel- and cobalt-based catalysts and petroleum sulfonates as emulsifiers at a lower reaction temperature of 180 °C. Chao et al. [13] used a 0.2% copper aromatic sulfonate composite catalyst to heat asphalt heavy oil at 280 °C for 24 h, and found that the viscosity of heavy oil decreased by 95.5%. Li et al. [14] designed an Fe^2+^ oil-soluble catalyst with toluenesulfonic acid as the ligand and reduced the viscosity of six heavy oil samples by 90% at 200 °C. The results show that the root cause of the decrease in the viscosity of crude oil is the depolymerization of asphaltenes in heavy oil. Suwaid et al. [15] used oil-soluble transition metal catalysts to catalyze the hydrothermal decomposition of heavy oils, and these catalysts had good catalytic performance at 300 °C, which could be used to improve the quality of heavy oil and reduce viscosity, among which nickel had the best catalytic performance. In addition, Chen et al. synthesized chromium alkylbenzenesulfonate and zinc alkylbenzenesulfonate, and its viscosity reduction rate was greater than 90%. Therefore, it is entirely feasible to reduce the viscosity of heavy oils using this method.

## 2. Results and Discussion

### 2.1. Infrared Analysis of Catalysts

**Figure 1:**
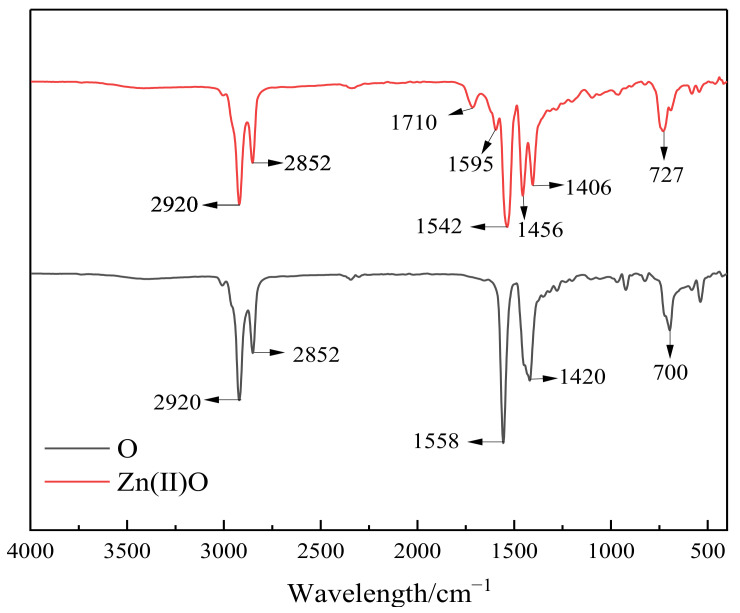
Infrared spectra of ligand O and catalyst Zn(II)O.

As shown in Figure 1, the oil-soluble catalyst Zn(II)O was analyzed by infrared spectroscopy. From Figure 1, it can be seen that there are two strong absorption peaks at 2800–2950 cm^−1^, which are the stretching vibration peaks of the C-H bond in methyl and methylene groups. The carboxyl groups of metal carboxylates have characteristic absorption peaks in two regions, with the absorption peak between 1610 and 1540 cm^−1^ being an anti-symmetric stretching vibration peak and the absorption peak between 1464 and 1362 cm^−1^ being a symmetric stretching vibration peak. Generally, the absorption peak intensity of the latter is weaker than that of the former [16]. The absorption peak at 1710 cm^−1^ is the stretching vibration peak of the C=O bond. Due to the inhibition of the conjugated structure of carboxylates by the ligand complexing with zinc ions, it is believed that the catalyst Zn(II)O has been generated.

### 2.2. Thermogravimetric Analysis of Catalysts

**Figure 2:**
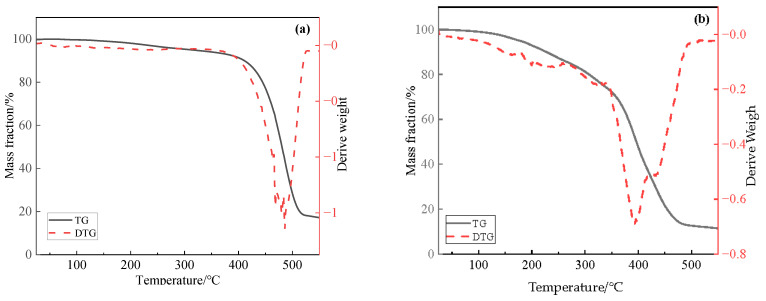
Thermogravimetric curves of ligand O (**a**) and oil-soluble catalyst Zn(II)O (**b**).

The thermogravimetric differential thermal analysis (TG-DTA) curves of catalyst Zn(II)O and ligand O are shown in Figure 2. Firstly, in the dehydration stage below 200 °C, the mass loss of ligand O is about 1.92%, and the mass loss of catalyst Zn(II)O is about 7.06%. In the second stage, between 200 and 350 °C, mainly due to the exothermic decomposition and oxidation of some substances, the mass loss of catalyst Zn(II)O in this stage is 20.65%. Finally, above 350 °C, both ligand O and catalyst Zn(II)O have strong exothermic peaks. In this stage, some gases are produced, mainly due to the decarboxylation of oleates. Among them, the weight loss rate of ligand O at 350–500 °C is 65.36%, and the mass loss of catalyst Zn(II)O is 59.64%. Moreover, because the complexation of ligand with zinc ion inhibits the conjugate structure of carboxylates, decarboxylation is easier to carry out, causing the peak time of the strong exothermic peak in the catalyst to shift from 485 °C to 392 °C. From the above analysis, it can be seen that the catalyst Zn(II)O was successfully synthesized.

### 2.3. Changes in Viscosity before and after Synergistic Catalytic Heavy Oil Reaction

**Figure 3:**
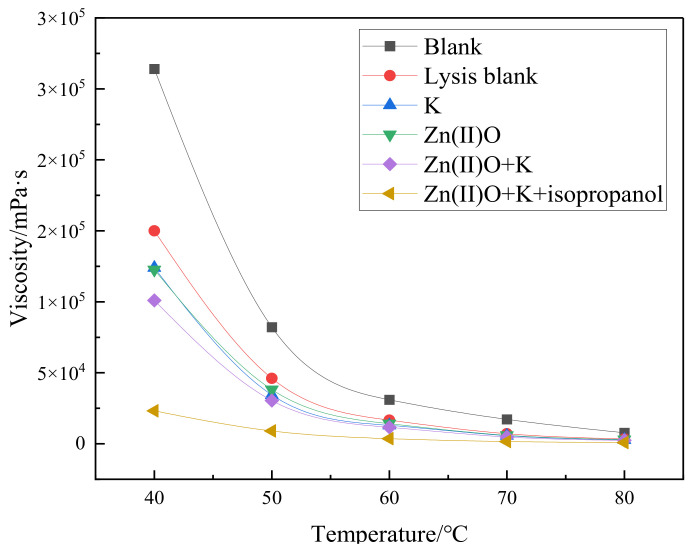
Viscosity–temperature curves of oil sample 1 before and after different reaction conditions.

**Figure 4:**
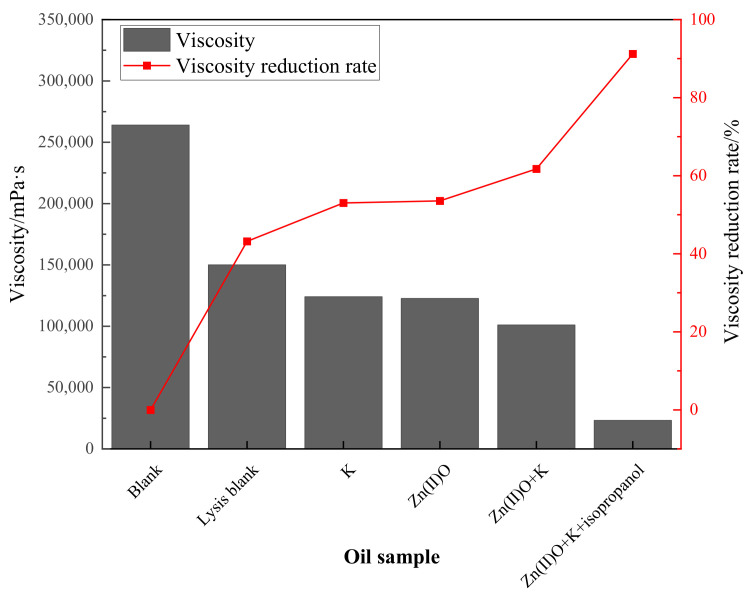
Effect of different reaction conditions on the viscosity and viscosity-reducing effect of oil sample 1.

From Figure 3, it can be seen that the viscosity reduction rate of heavy oil after pure hydrothermal cracking is 43.18%. The addition of reservoir minerals increases the viscosity reduction rate by 9.85% on the basis of the cracking blank, and the addition of oil-soluble catalyst Zn(II)O increases the viscosity reduction rate by 10.38% on the basis of the cracking blank. After the synergistic reaction of Zn(II)O + K, the reservoir minerals achieve catalytic effect by exchanging ions with external catalysts, resulting in an 18.56% increase in viscosity reduction compared to the cracking blank. Compared with the separate catalysis of Zn(II)O and reservoir minerals, the viscosity reduction effect is further improved. From Figure 4, it can be seen that after being catalyzed by Zn(II)O + K + isopropanol, the viscosity reduction rate further increased by 41.37% compared to the cracking blank.

### 2.4. Evaluation of Viscosity Reduction Stability and Universality of Catalysts

**Figure 5:**
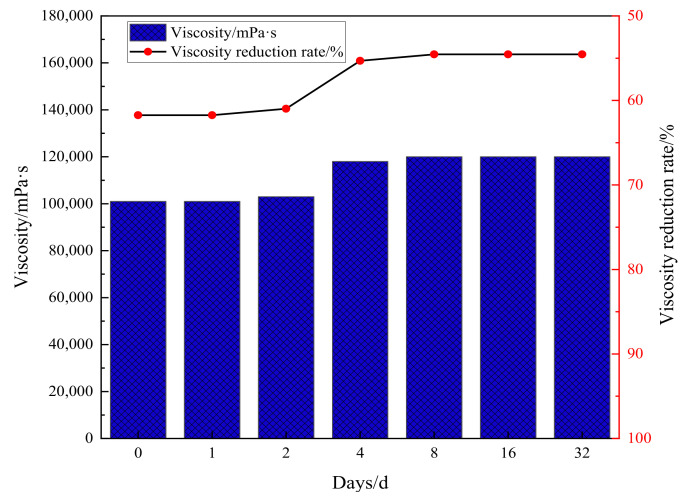
Evaluation of Zn(II)O + K synergistic effect on viscosity-reducing stability of oil sample 1.

**Figure 6:**
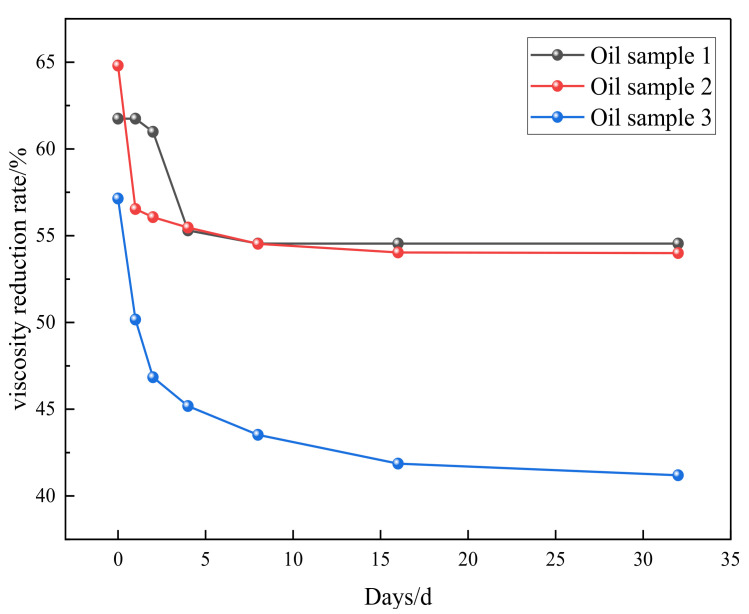
Universal evaluation of Zn(II)O + K synergistic effect on different oil samples.

As shown in Figure 5, the viscosity of heavy oil under synergistic catalysis will rebound in a short period of time after hydrothermal cracking, and the fluctuation range of viscosity will be small afterwards. The viscosity reduction rate remains stable at around 55%, and the synergistic catalytic stability of Zn(II)O + K on oil sample 1 is good. As shown in Figure 6, the universality evaluation of Zn(II)O + K on different oil samples shows that the viscosity of different oil samples rebounded significantly in a short period of time, and then the viscosity reduction rate reached a stable level. This is mainly due to the re-establishment of intermolecular forces such as hydrogen bonds in the groups containing heteroatoms in the heavy oil, which caused the previously formed smaller molecules to become larger molecules again. In addition, the re-entanglement of alkyl chains led to an increase in viscosity, After the intermolecular interaction stabilizes, there is no significant change in viscosity. From this analysis, it can be seen that the catalytic effect of Zn(II)O + K can achieve a viscosity reduction rate of over 40% for all three types of oil samples, which can prove that the synergistic catalytic viscosity reduction effect of Zn(II)O + K is relatively stable.

### 2.5. Changes in Pour Point of Heavy Oil before and after Reaction

**Figure 7:**
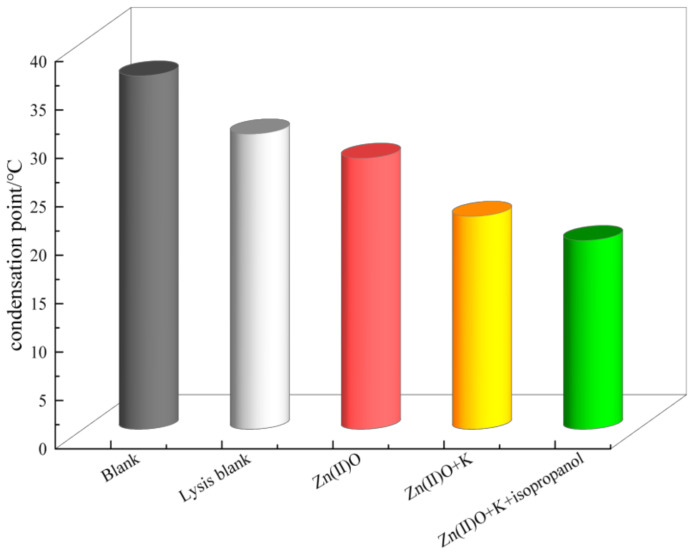
Solidification points of heavy oil before and after different reaction systems.

As shown in Figure 7, the blank oil sample has a high pour point, and after hydrothermal cracking, the pour point decreases significantly, from 36.5 °C to 30.5 °C, with a decrease of up to 6 °C. After the synergistic catalysis of Zn(II)O + K, the pour point of the oil sample decreased to 22 °C, with a decrease of 8.5 °C. And after being catalyzed by the hydrogen donor isopropanol, the condensation point further decreased to 18.5 °C.

### 2.6. Thermogravimetric Analysis of Heavy Oil before and after Reaction

**Figure 8:**
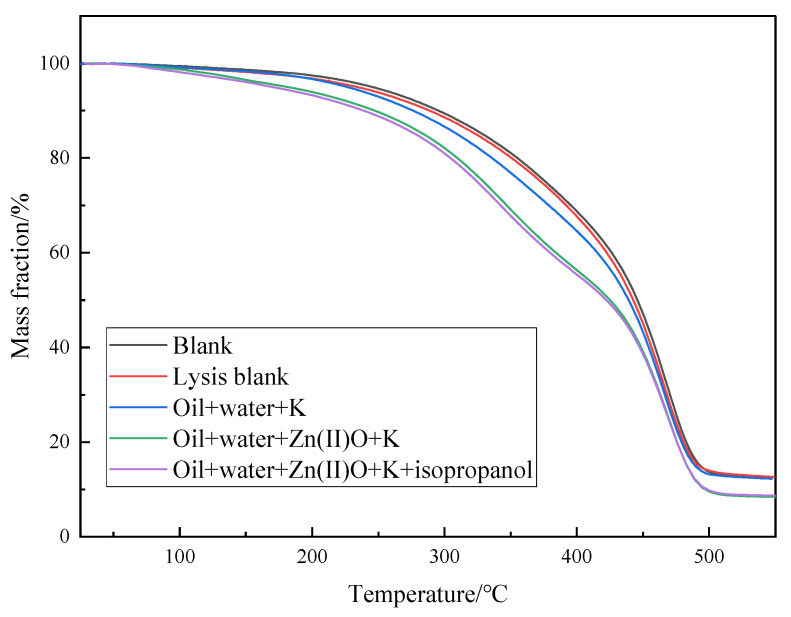
Thermogravimetric curves of oil sample 1 before and after hydrothermal cracking reaction.

Shown in Figure 8 is the thermogravimetric analysis of oil sample 1 before and after different reaction systems. The pyrolysis process of heavy oil can be divided into three stages. First, in the temperature range of 25–150 °C, the volatilization of low-carbon hydrocarbons and water is carried out, and the weight loss of blank, oil + water, oil + water + K, oil + water + Zn(II)O + K, and oil + water + Zn(II)O + K + isopropanol is 2.58%, 3.19%, 3.33%, 6.11%, and 6.81%, respectively. The second stage is in the temperature range of 150–350 °C. According to the research, the volatilization of light components takes place, as well as and the fracture of weak chemical bonds during the process and the low degree of cracking of soft asphalt, in which the weight loss rates of oil samples are 16.27%, 16.66%, 19.94%, 26.62%, and 24.62%, respectively, indicating that some asphaltenes are converted into low-carbon hydrocarbons after hydrothermal cracking, and the further volatilization of low-carbon hydrocarbons during thermogravimetric analysis leads to an increase in weight loss rate. In the third stage, the temperature range is between 350 and 550 °C, and the weight loss rates of the oil samples are 68.50%, 67.55%, 64.50%, 58.83%, and 59.84%, respectively. This is mainly due to the high cracking of heavy components in the heavy oil. From Figure 8 and the above analysis, it can be seen that compared to the blank oil sample, the weight loss curves of different reaction systems all shift to the left, which also reversely proves that the recombination splitting decomposes into some light components, thereby reducing the viscosity of heavy oil.

### 2.7. DSC Analysis of Heavy Oil before and after Reaction

**Figure 9:**
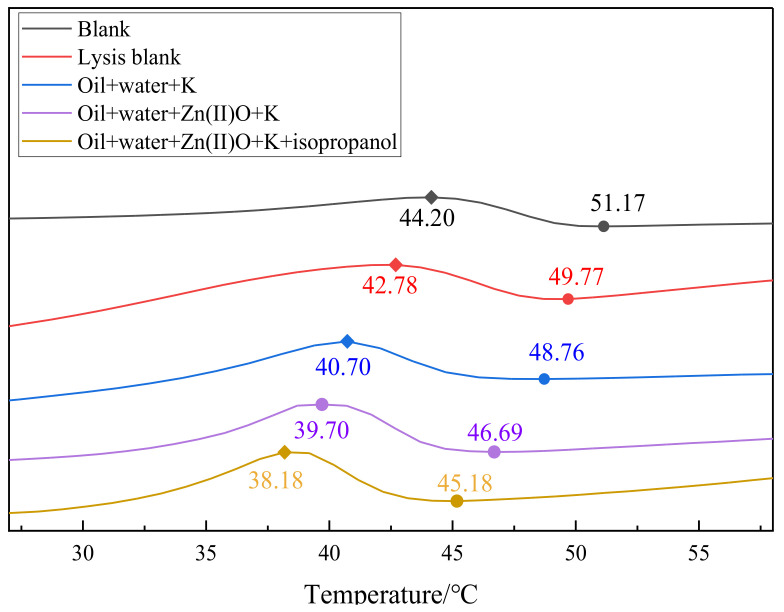
DSC curve of oil sample 1 before and after hydrothermal cracking reaction.

As shown in Figure 9, the wax precipitation point of blank sample 1 is 51.17 °C. After hydrothermal cracking, the wax precipitation point decreases to 49.77 °C, and the peak temperature of wax precipitation also decreases from 44.20 °C to 42.78 °C of the blank sample. After adding oil, water, and K for the hydrothermal cracking reaction, the wax precipitation point decreases to 48.76 °C. After the synergistic reaction of oil + water + Zn(II)O + K, the wax precipitation point decreased to 46.69 °C, which was 4.48 °C lower than that of the blank oil sample. After adding isopropanol into the reaction system, the wax precipitation point moved left to 45.18 °C, and the wax precipitation peak temperature significantly shifted left, decreasing from 44.20 °C to 38.18 °C in the blank oil sample. From the above analysis of results, it can be seen that some of the heavy components in the oil sample are decomposed into light components. At the same time, some of the light components generated will act as solvents, dissolve some of the heavy components, and thus reduce the precipitation temperature of wax crystals.

### 2.8. Element Analysis and Four-Component Analysis of Heavy Oil before and after Reaction

**Figure 10:**
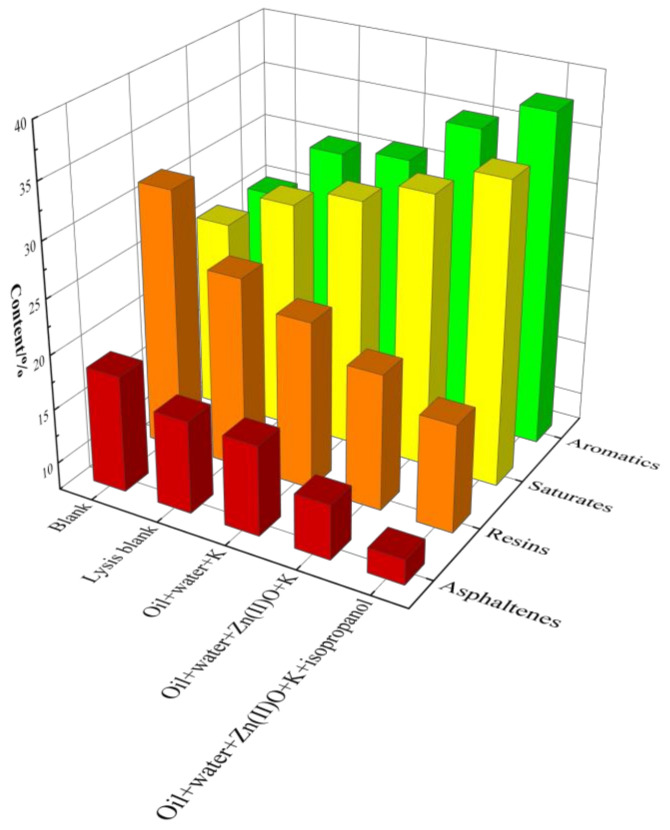
Component analysis of oil sample 1 before and after hydrothermal cracking reaction.

The four-component analysis of oil samples after reaction in different reaction systems is shown in Figure 10. Without a catalyst, the content of saturated and aromatic hydrocarbons in the oil sample increased after hydrothermal cracking, while the content of resin and asphaltene decreased. Among them, Zn(II)O + K synergistic catalysis has high catalytic performance, and the content of saturated hydrocarbons, aromatic hydrocarbons, resins, and asphaltenes in the oil sample after reaction is 32.35%, 35.14%, 19.98%, and 12.53%, respectively. After being catalyzed by the hydrogen donor isopropanol, the content of saturated and aromatic hydrocarbons in the oil sample further increased to 34.96% and 35.75%, respectively, and the content of resin and asphalt also significantly decreased, reaching 19.95% and 10.37%, respectively. From the above analysis, it can be seen that the synergistic catalytic reaction between external catalysts and reservoir minerals can indeed increase the light component content of heavy oil.

The elemental analysis results of oil samples after different reaction systems are shown in Table 1. The C/H ratios of the blank oil sample, the oil sample catalyzed by Zn(II)O + K, and the oil sample catalyzed by Zn(II)O + K + isopropanol were 8.54, 8.15, and 8.13, respectively, indicating a significant decrease in C/H ratios. This may be due to the catalyst promoting the hydrogenation reaction of unsaturated bonds [17]. After adding Zn(II)O + K and Zn(II)O + K + isopropanol as catalysts, the content of C, N, and S in the oil sample decreased to varying degrees, while the content of H increased. This may be due to the cleavage of the C-X (X: N, S, O) bond and the involvement of water in the catalytic hydrothermal cracking of heavy oil [18,19,20], resulting in a significant decrease in the viscosity of heavy oil.

### 2.9. GC Analysis of Saturated Hydrocarbon Components in Heavy Oil before and after Reaction

**Figure 11:**
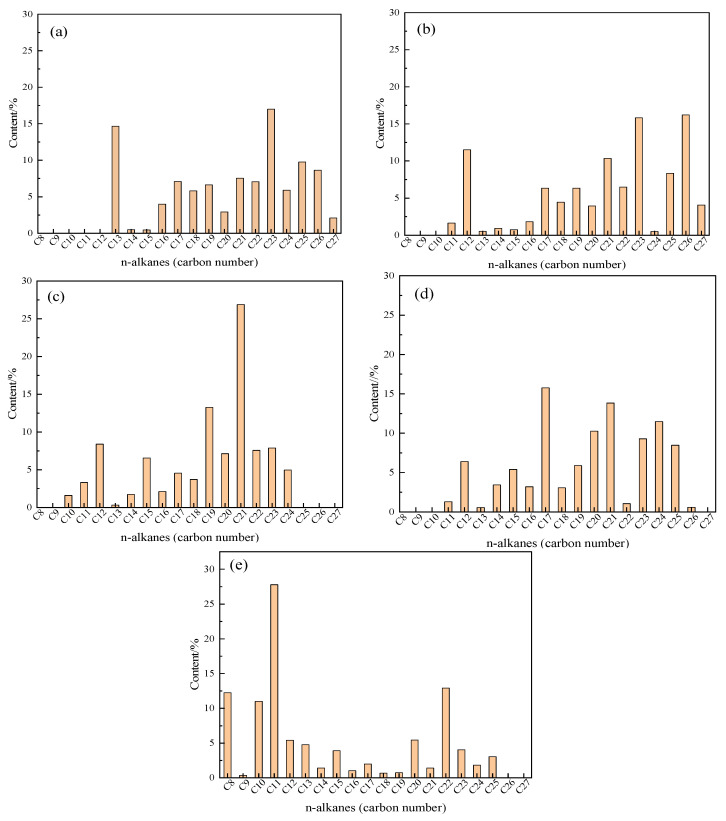
Carbon number distribution of oil samples before and after different reaction systems (**a**): Blank; (**b**): Oil + water; (**c**): Oil + water + K; (**d**): Oil + water + Zn(II)O + K; (**e**): Oil + water + Zn(II)O + K + isopropanol.

Figure 11 show that the carbon number of the blank oil sample is mostly concentrated in the high carbon number part, and the content of oil and water in the sample increases significantly at C_11_ and C_12_ compared to the blank. The carbon number of oil sample + water + K is mostly concentrated in C_19_-C_21_. After the synergistic reaction, the carbon number distribution of oil sample oil + water + Zn(II)O + K significantly shifts forward, and the distribution is relatively uniform. After adding the additive isopropanol to the reaction system, the high carbon number of the oil sample significantly decreased, and the content of low carbon number significantly increased, with C_8_, C_10_, and C_11_ content increasing more significantly. The different distribution of carbon number content directly represents the different changes in saturated hydrocarbon content in heavy oil, and the difference in saturated hydrocarbon content directly affects the pour point of heavy oil. This analysis result contradicts the change in oil sample pour point and the phenomenon of left shift in the DSC thermal analysis results.

### 2.10. Analysis of Wax Crystal Morphology before and after Heavy Oil Reaction

**Figure 12:**
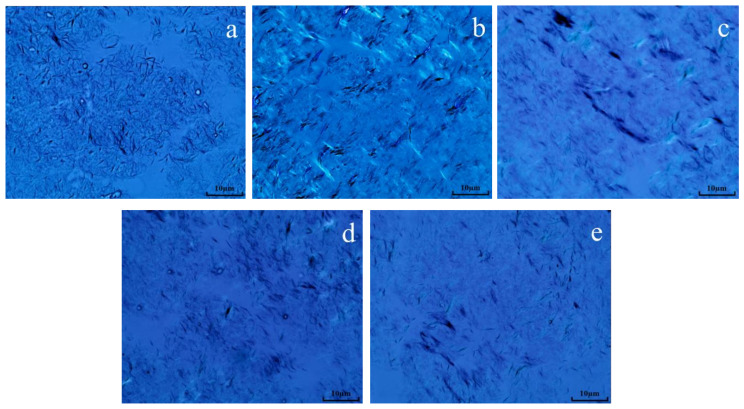
Wax crystal morphology of oil sample 1 before and after reaction ((**a**): blank; (**b**): cracking blank; (**c**): oil + water + K; (**d**): oil + water + Zn(II)O + K; (**e**): Oil + water + Zn(II)O + K + isopropanol).

The morphology of wax crystals in oil sample 1 before and after reaction was analyzed using a polarizing microscope. From Figure 12, it can be seen that the wax crystal morphology in the oil sample before hydrothermal cracking was significantly stacked, presenting a snowflake shape. After hydrothermal cracking, it was found that the wax crystal stacking structure was significantly reduced, showing mostly needle-like stacking. After the reaction of oil + water + K, the aggregation of wax crystals is significantly reduced, and the degree of wax crystal dispersion increases. After the synergistic catalytic reaction of oil + water + Zn(II)O + K, the degree of wax crystal dispersion significantly increases, and the wax crystal morphology of the oil sample is more sparse compared to the oil sample catalyzed by a single catalyst. After the reaction of oil + water + Zn(II)O + K+ isopropanol, the degree of dispersion of the oil sample further increases, and isopropanol itself plays a role in dissolution and dilution. The main reason for the increase in wax crystal dispersion may be the cracking of colloidal asphaltene macromolecules in heavy oil into low-carbon hydrocarbons, which act as solvents, leading to a decrease in the solidification point and wax precipitation point after cracking, further dispersing the wax crystal structure and improving the fluidity of heavy oil [21,22,23].

### 2.11. GC-MS Analysis of Heavy Oil Aqueous Phase before and after Reaction

**Figure 13:**
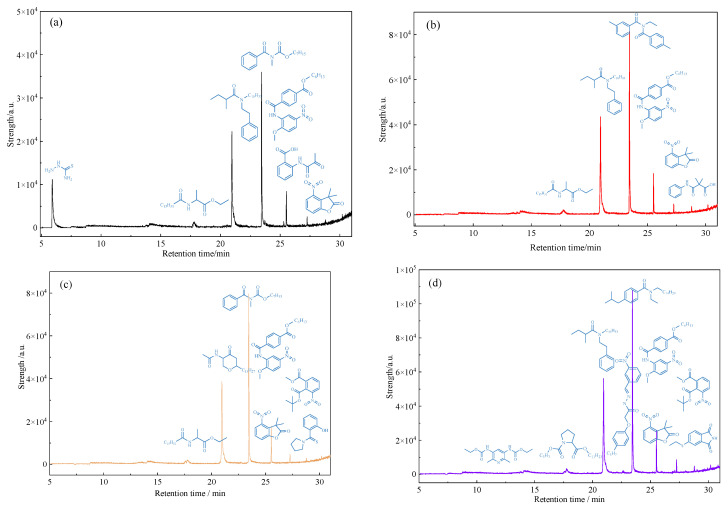
GC-MS analysis of polar substances dissolved in water after reaction of oil sample 1 (**a**): Oil + water; (**b**): Oil + water + K; (**c**): Oil + water + Zn(II)O + K; (**d**): Oil + water + Zn(II)O + K + isopropanol.

GC-MS was used to analyze the treated water phase after the reaction, and the analysis results are shown in Figure 13. From the analysis of Figure 13, it can be seen that after the oil sample is subjected to hydrothermal cracking and catalytic reaction treatment, polar organic compounds containing heteroatoms will dissolve in water. The types of polar substances and heteroatom-containing substances in water have undergone significant changes, and the types of heteroatom-containing substances in water have increased. This fully indicates that after hydrothermal cracking, the chemical bond of the glial asphaltene and other components in the heavy oil will break after cracking, which will significantly reduce the viscosity of the heavy oil, and a small part of the heteroatomic organic matter generated after the bond breaking will enter the water phase components.

## 3. Mechanism

### 3.1. Catalytic Aquathermolysis of Model Compounds

The catalytic hydrothermal cracking of heavy oil by model compounds mainly involves the reaction between asphaltene and resin, which are macromolecules containing five-membered and six-membered rings with heteroatoms and alkyl branching. Based on its chemical structure, model compounds representing its special chemical structure were selected for catalytic hydrothermal decomposition research to explore its viscosity reduction mechanism, choice of α- seven model compounds, octene, nonylphenol, and benzothiophene, and separate the liquid phase for subsequent GC-MS analysis.

**Figure 14:**
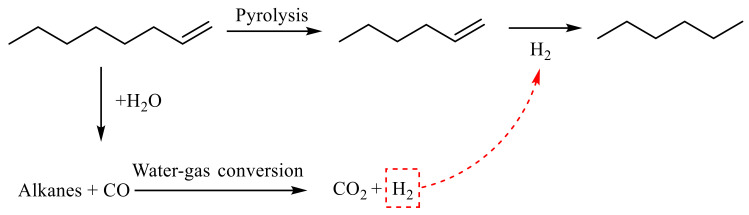
α-Octene reaction mechanism.

As shown in Table 2 showing the α-compounds, after the reaction of octene, n-hexene was generated at 2.761 min. During the reaction, octene was cracked to produce low-carbon olefins, such as n-hexene in the analysis. The fallen short-chain alkanes underwent water gas conversion during the hydrothermal cracking process, resulting in further reaction to generate CO_2_ and H_2_. At 3.094 min, n-hexane was generated, and the hydrogenation reaction of n-hexene generated in the previous step generated n-hexane. Based on the GC-MS analysis results and combined with [24,25,26,27,28], it is inferred that the reaction path of octene cracking is shown in Figure 14.

**Figure 15:**
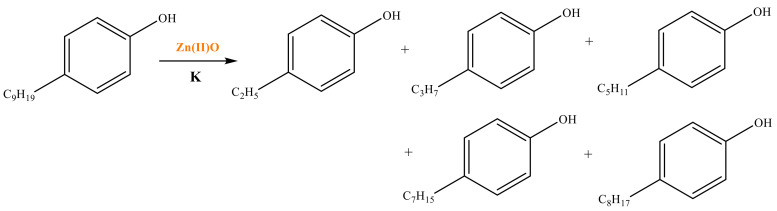
Reaction mechanism of nonylphenol.

As shown in Table 3, there are many peaks at 26 min, which are the characteristic peaks of nonylphenol. Under different reaction conditions, nonylphenol mainly undergoes C-C bond cleavage and recombination, resulting in the formation of different compounds. The different alkyl short chains that fall off combine with -OH to form alcohols during hydrothermal processes and undergo oxidation reactions at high temperatures to generate CO_2_. Based on the GC-MS analysis results and literature review [29,30], the mechanism of the hydrothermal cracking reaction of nonylphenol was derived, as shown in Figure 15.

**Figure 16:**
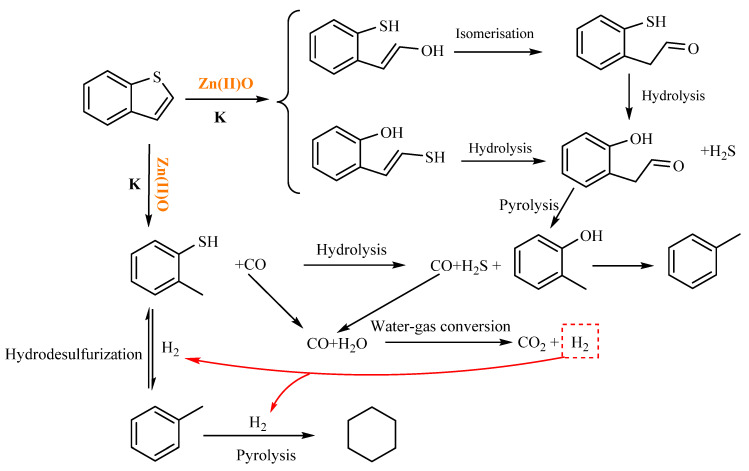
Reaction mechanism of benzothiophene.

As shown in Table 4, cyclohexane is generated at 2.761 min after the reaction of benzothiophene, and cyclohexane is generated after catalytic thermal cracking, hydrodesulfurization, and catalytic hydrogenation of benzothiophene. Toluene was generated at 5.600 min. According to the literature review, o-methylphenol was hydrogenated and deoxygenated to form toluene. Based on the results of GC-MS analysis and [23,31], the mechanism of the hydrothermal cracking reaction of benzothiophene was derived, as shown in Figure 16.

### 3.2. Catalytic Viscosity Reduction Mechanism

**Figure 17:**
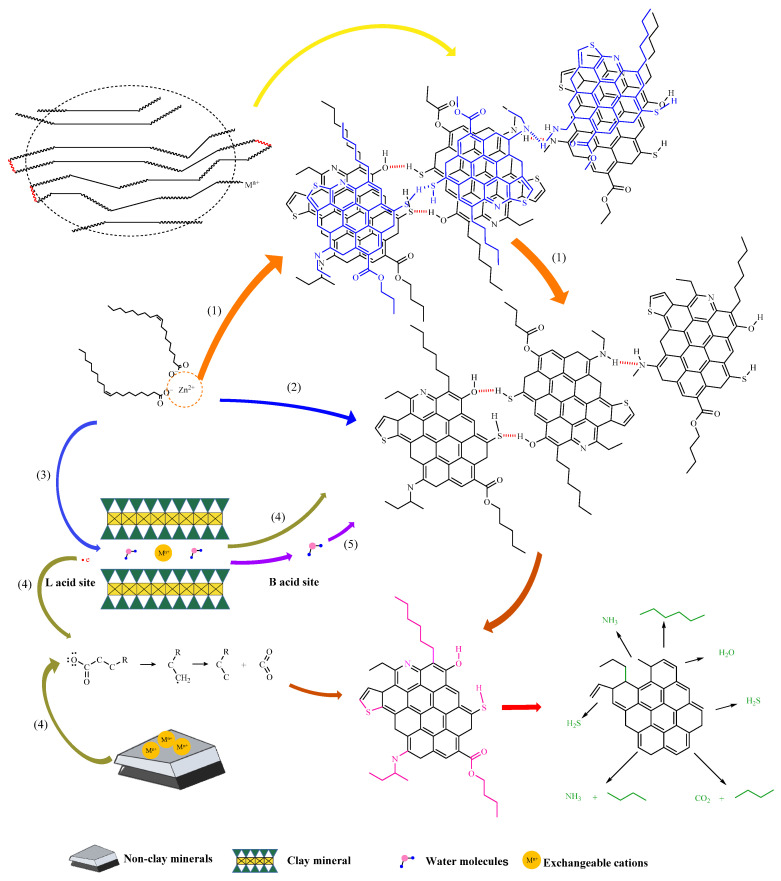
Synergistic catalytic mechanism of exogenous catalysts and reservoir minerals.

The synergistic dual catalytic mechanism between exogenous catalysts and reservoir minerals is shown in Figure 17, which is mainly divided into the following stages:

(1) Due to the high content of glial asphaltene in heavy oil, there is a huge van der Waals force between layer units, leading to mutual association between units, which is intuitively manifested as high viscosity and poor fluidity. The addition of exogenous catalysts greatly affects or destroys the active site, resulting in partial permanent depolymerization and partial loose binding. As a result, some flaky units are depolymerized and separated, and the viscosity of heavy oil is significantly reduced.
(2) The external catalyst acts on the heteroatoms in the recombination component, disrupting the hydrogen bonds between some high-carbon hydrocarbon compounds, resulting in the cleavage of C-S, C-O, and C-N bonds.
(3) The surface of reservoir minerals is negatively charged due to the substitution effect of the lattice, thereby adsorbing cations, enabling reservoir minerals to function as normal catalysts and carriers. The transition metals in the exogenous catalyst are easily exchanged with sodium/calcium ions in the clay, thus becoming the active centers in the reaction. Due to the presence of a large number of empty orbitals, transition metals can easily interact with electron-rich substances in heavy oil, greatly improving the catalytic effect of hydrothermal cracking [32].
(4) Under high temperature, clay minerals act as strong acid, and the catalytic mechanism of mineral matrix-forming oil and gas is the carbonium ion mechanism, that is, the acid center on the surface of the mineral matrix can promote kerogen to form a carbonium ion, and the catalytic effect is achieved through the decomposition and transfer of the carbonium ion [33,34]. Quartz, calcite, and other components in non-clay minerals will absorb free cations to form L-acid, which is also conducive to the transformation of kerogen [35,36]. Due to the presence of Lewis acid on the surface of minerals, high-carbon hydrocarbon compounds provide electrons and generate free radicals, which rearrange and promote the cleavage of C-C bonds, forming short-chain alkanes. Clay minerals act as B acid, providing proton H+ for adsorbed organic matter. Proton (H+) comes from the dissociation of adsorbed water and interlayer water molecules combined with exchangeable cations, which mainly react by forming transition state carbonium ions [37,38].
(5) When water molecules are adsorbed on the surface of clay minerals, because L-acid has a strong affinity for electrons, it can share a pair of electrons with the hydroxyl group in water, making the hydroxyl group firmly adsorbed on the surface of L-acid, and the remaining H + is easy to release, which will convert L-acid into B-acid. When clay minerals are dehydrated, due to the lack of protons, B acid sites are gradually transformed into L acid [39,40,41]. In this reaction system, clay minerals activate the reactant water/steam, reduce the reaction activation energy, accelerate the fracture speed of some hydrogen bonds between the molecules of high-carbon hydrocarbon compounds, and improve the efficiency of reducing the viscosity of heavy oil.

## 4. Materials and Methods

### 4.1. Preparation and Naming of Oil-Soluble Exogenous Catalysts

**Figure 18:**
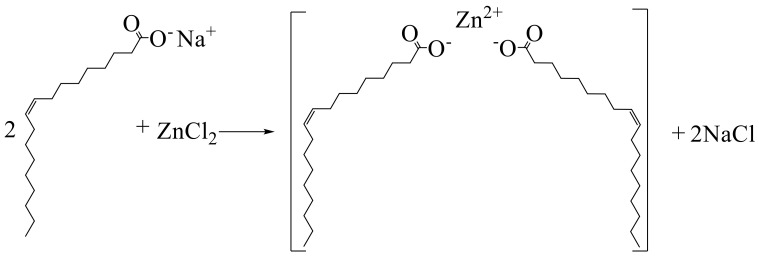
Reaction mechanism of Zn(II)O.

We used a zinc chloride and ligand sodium oleate (O) massage ratio of 1:3 dissolved in the mixture of deionized water, ethanol, and n-hexane (mixed solution ratio 1:1:1). The mixture was heated at 70 °C reflux for 4 h, transferred to the separation funnel, and the upper organic layer containing the complex was washed with water. After washing the upper organic layer at 60 °C, vacuum drying was carried out for 15 h. The resulting product is named Zn(II)O, and the reaction mechanism is shown in Figure 18.

### 4.2. Oil Sample and Physical Properties

In this experiment, the oil samples used were from Henan Oilfield oil sample (later called oil sample 1), Henan Nanyang Oilfield oil sample (later called oil sample 2), and Henan Tanghe oilfield oil sample (later called oil sample 3). The properties of heavy oils are shown in Table 5. The reagents used in the experiment are analytically pure and do not require further processing.

### 4.3. Performance Evaluation

Take a certain amount of oil sample and put it in a water bath at 65 °C, take 30 g in the reactor after constant temperature for 1 h, and then add the prepared oil-soluble catalyst to the reactor according to its ratio of 0.2% to the oil mass, vacuum it, fill with nitrogen, react at 180 °C for 4 h, add a certain amount of water according to the water–oil mass ratio of 30%, add the catalyst according to the catalyst-to-oil mass ratio of 0.2%, reduce to room temperature after the end of the reaction, centrifuge, put it in a measuring cup, and subsequently measure its viscous temperature properties and other physical properties.

### 4.4. Characterization before and after the Heavy Oil Water Thermal Cracking Reaction

Viscosity of heavy oils is measured according to ASTM D97-96. The viscosity reduction rate of oil, Δη%, was calculated from ((η0 − η)/η0) × 100, where η0 and η (mPa∙s) are, respectively, the viscosities of the oil before and after the reaction [7]. Furthermore, the analysis of heavy oil components was carried out in accordance with the China Petroleum Industry Standard SY/T 5119-2016 [42]. The elemental compositions (C, H, N, and S) of initial oil and upgraded oil were measured by elementar vario EL cube. Determination of the four components of petroleum asphaltene was performed according to standard NB/SH/T0509-2010 [43]. Thermogravimetric analysis is used to evaluate the distribution of carbon number in crude oil in different temperature ranges. The oil samples were heated from 30 °C to 550 °C under nitrogen atmosphere at a heating rate of 10 °C/min. The wax precipitation point of heavy oil was measured according to SY/T 0545-2012 [44]. The different scanning calorimetry (DSC) analyses of heavy oil were all determined using the instrument Mettler-Toledo DSC822e DSC (Greifensee, Switzerland) under a nitrogen atmosphere with a flow rate of 50 mL/min and a temperature range of −25 to 50 °C. Microstructural analysis of wax crystals was performed using saturated hydrocarbons separated from crude oil, and observed at 15 °C (±0.2 °C) using a polarizing microscope (BX41-OLYMPUS). The composition analysis of the model compound was measured via GC-MS using a 7890A-5975C (Santa Clara, CA, USA), with hydrogen as a carrier gas, keeping the flow rate at 25 mL/min. The composition analysis of the samples was identified by using the DRS compound database.

## 5. Conclusions

The synergistic catalytic effect of oil-soluble exogenous catalysts and reservoir minerals in heavy oil hydrothermal cracking was studied. The viscosity reduction effect of Zn(II)O + K is the best. Compared with the blank of oil sample 1 at 40 °C, its viscosity reduction rate can reach 61.74%, and compared with the cracking blank, its viscosity reduction rate can reach 32.67%. After being catalyzed by isopropanol, the viscosity reduction rate further increased by 41.37% compared to the cracking blank, reaching 91.22%, thereby reducing the viscosity of heavy oil. After the catalytic hydrothermal decomposition of heavy oil, the results of thermogravimetric and DSC analysis showed that the heavy components decreased and the content of light components increased.

## Figures and Tables

**Table 1:** Element analysis results of oil samples before and after reaction.

Oil Sample	C/%	H/%	N/%	S/%	C/H
Blank	86.22	10.10	2.25	0.45	8.54
Lysis blank	85.08	10.06	2.17	0.39	8.46
Oil + water + K	84.11	10.01	2.15	0.35	8.40
Oil + water + Zn(II)O + K	83.54	10.25	1.82	0.29	8.15
Oil + water + Zn(II)O + K + isopropanol	83.45	10.26	1.21	0.28	8.13

**Table 2:** α-Compound after octene reaction.

Compound Structural Formula α-Octene + Water	Compound Structural Formula α-Octene + Water + K	Compound Structural Formula α-Octene + Water + Zn(II)O + K	Compound Structural Formula α-Octene + Water + Zn(II)O+ K+ Isopropanol
		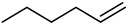	
		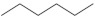	
		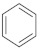	
		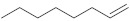	

**Table 3:** Compounds after nonylphenol reaction.

Compound Structural Formula Nonylphenol + Water	Compound Structural Formula Nonylphenol + Water + K	Compound Structural Formula Nonylphenol + Water + Zn(II)O+K	Compound Structural Formula Nonylphenol + Water + Zn(II)O+K + Isopropanol
		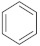	
		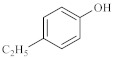	
		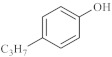	
		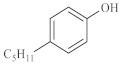	
			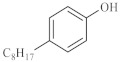
		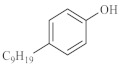	
		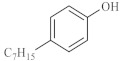	

**Table 4:** Compounds after benzothiophene reaction.

Compound Structural Formula Benzothiophene + Water	Compound Structural Formula Benzothiophene+ Water + K	Compound Structural Formula Benzothiophene + Water+ Zn(II)O + K	Compound Structural Formula Benzothiophene + Water + Zn(II)O + K+ Isopropanol
		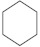	
		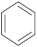	
		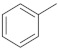	
		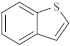	

**Table 5:** Main physical parameters of heavy oil.

Heavy Oil	Pour Point (°C)	Water Content (%)	Saturates (%)	Aromatics (%)	Resins (%)	Asphaltenes (%)
Oil sample 1	38.0	15.5	25.26	33.98	25.55	15.21
Oil sample 2	20.0	17.0	31.16	28.73	16.67	23.44
Oil sample 3	19.6	12.5	24.76	31.28	18.57	25.39

## Data Availability

The data presented in this study are available wholly within the manuscript.

## References

[1] Fan Z.-X., Wang T.-F., He Y.-H. (2009). Upgrading and viscosity reduction of heavy oil by catalyst ionic liquid. J. Fuel Chem. Technol..

[2] Li Y., Liu J., Li W., Dou M., Ma L., Wang Q., Zhao B., Chen G. (2023). Enhanced sorption for the oil spills by SDS-modified rice straw. Gels.

[3] Iskandar F., Dwinanto E., Abdullah M., Khairurrijal, Muraza, O (2016). J.P. Viscosity Reduction of Heavy Oil Using Nanocatalyst in Aquathermolysis Reaction. KONA Powder Part. J..

[4] Jia C., Zheng M., Zhang Y. (2012). Unconventional hydrocarbon resources in China and the prospect of exploration and development. Pet. Explor. Dev..

[5] Ma L., Slaný M., Guo R., Du W., Li Y., Chen G. (2023). Study on synergistic catalysis of ex-situ catalyst and in-situ clay in aquathermolysis of water-heavy oil-ethanol at low temperature. Chem. Eng. J..

[6] Li Y., Bai Q., Li Q., Huang H., Ni W., Wang Q., Xin X., Zhao B., Chen G. (2023). Preparation of Multifunctional Surfactants Derived from Sodium Dodecylbenzene Sulfonate and Their Use in Oil-Field Chemistry. Molecules.

[7] Kuzmić A.E., Radošević M., Bogdanić G., Srića V., Vuković R. (2008). Studies on the influence of long chain acrylic esters polymers with polar monomers as crude oil flow improver additives. Fuel.

[8] Muraza O., Galadima A. (2015). Aquathermolysis of heavy oil: A review and perspective on catalyst development. Fuel.

[9] Huang S., Cao M., Huang Q., Liu B., Jiang J. (2019). Study on reaction equations of heavy oil aquathermolysis with superheated steam. Int. J. Environ. Sci. Technol..

[10] Zhao F., Liu Y., Fu Z., Zhao X. (2014). Using hydrogen donor with oil-soluble catalysts for upgrading heavy oil. Russ. J. Appl. Chem..

[11] Liu Z., Bai B., Tang J., Xiang Z., Zeng S., Qu H. (2021). Investigation of slickwater effect on permeability of gas shale from longmaxi formation. Energy Fuel.

[12] Zhao X.-F., Tan X.-H., Liu Y.-J. (2008). Behaviors of oil-soluble catalyst for aquathermolysis of heavy oil. Ind Catal.

[13] Chao K., Chen Y., Li J., Zhang X., Dong B. (2012). Upgrading and visbreaking of super-heavy oil by catalytic aquathermolysis with aromatic sulfonic copper. Fuel Process. Technol..

[14] Li J., Chen Y., Liu H., Wang P., Liu F. (2013). Influences on the aquathermolysis of heavy oil catalyzed by two different catalytic ions: Cu^2+^ and Fe^3+^. Energy Fuel.

[15] Suwaid M.A., Varfolomeev M.A., Al-Muntaser A.A., Yuan C., Starshinova V.L., Zinnatullin A., Vagizov F.G., Rakhmatullin I.Z., Emelianov D.A., Chemodanov A.E. (2020). In-situ catalytic upgrading of heavy oil using oil-soluble transition metal-based catalysts. Fuel.

[16] Luo M., Guan P., Liu W.-H. (2007). The identification of several saturated fatty acids and their salts by means of infrared spectrometry. Guang Pu Xue Yu Guang Pu Fen Xi=Guang Pu.

[17] Chao K., Chen Y., Liu H., Zhang X., Li J. (2012). Laboratory experiments and field test of a difunctional catalyst for catalytic aquathermolysis of heavy oil. Energy Fuel.

[18] Al-Muntaser A.A., Varfolomeev M.A., Suwaid M.A., Feoktistov D.A., Yuan C., Klimovitskii A.E., Gareev B.I., Djimasbe R., Nurgaliev D.K., Kudryashov S.I. (2021). Hydrogen donating capacity of water in catalytic and non-catalytic aquathermolysis of extra-heavy oil: Deuterium tracing study. Fuel.

[19] Shokrlu Y.H., Babadagli T. (2014). Viscosity reduction of heavy oil/bitumen using micro-and nano-metal particles during aqueous and non-aqueous thermal applications. J. Pet. Sci. Eng..

[20] Liu Z., Bai B., Wang Y., Qu H., Ding Z., Xiao Q. (2021). Experimental study of slickwater volume effect on methane desorption on Longmaxi shale. J Nat Gas Sci Eng.

[21] Fan H.-F., Liu Y.-J., Zhong L.-G. (2001). Studies on the synergetic effects of mineral and steam on the composition changes of heavy oils. Energy Fuel.

[22] Cao H., Cao X., Zhao X., Guo D., Liu Y., Bian J. (2022). Molecular dynamics simulation of wax molecules aggregational crystallization behavior during cooling of crude oil mixture. Case Stud. Therm. Eng..

[23] Bian J., Ding G., Guo D., Cao H., Liu Y., Cao X. (2023). Surface crystallization mechanism of n-hexane droplets. Energy.

[24] Chen Y., He J., Wang Y., Li P. (2010). GC-MS used in study on the mechanism of the viscosity reduction of heavy oil through aquathermolysis catalyzed by aromatic sulfonic H_3_PMo_12_O_40_. Energy.

[25] Dong S., Li H., Bloede I.K., Al Abdulghani A.J., Lebrón-Rodríguez E.A., Huber G.W., Hermans I. (2023). Catalytic conversion of model compounds of plastic pyrolysis oil over ZSM-5. Appl. Catal. B Environ..

[26] Boulet P., Greenwell H., Stackhouse S., Coveney P. (2006). Recent advances in understanding the structure and reactivity of clays using electronic structure calculations. J. Mol. Struct. THEOCHEM.

[27] Wu L.M., Zhou C.H., Keeling J., Tong D.S., Yu W.H. (2012). Towards an understanding of the role of clay minerals in crude oil formation, migration and accumulation. Earth-Sci. Rev..

[28] Guo D., Cao X., Ma L., Zhang P., Liu Y., Bian J. (2023). Bulk and interfacial properties of methane-heavy hydrocarbon mixtures. Energy.

[29] Huandi H., Yi Z., Ming D., Haiping S., Zhenyu D., Jun L. (2021). Study on thermal cracking reaction of thiophene model compounds in the presence of hydrogen and dispersed catalyst. Acta Pet. Sin. (Pet. Process. Sect.).

[30] Canıaz R.O., Arca S., Yaşar M., Erkey C. (2019). Refinery bitumen and domestic unconventional heavy oil upgrading in supercritical water. J. Supercrit. Fluids.

[31] Zeshi Z., Xiaowa N., Chunshan S., Xinwen G. (2021). Theoretical Study on Hydrodeoxygenation of o-Cresol Over Pd-Doped Fe Catalyst. Acta Pet. Sin. (Pet. Process. Sect.).

[32] Cornejo J., Celis R., Pavlovic I., Ulibarri M. (2008). Interactions of pesticides with clays and layered double hydroxides: A review. Clay Miner..

[33] Karpiński B., Szkodo M. (2015). Clay minerals–mineralogy and phenomenon of clay swelling in oil & gas industry. Adv. Mater. Sci..

[34] Zhao K., Wang X., Pan H., Li Q., Yang J., Li X., Zhang Z. (2018). Preparation of molybdenum-doped akaganeite nano-rods and their catalytic effect on the viscosity reduction of extra heavy crude oil. Appl. Surf. Sci..

[35] Al-Otoom A.Y., Shawabkeh R.A., Al-Harahsheh A.M., Shawaqfeh A.T. (2005). The chemistry of minerals obtained from the combustion of Jordanian oil shale. Energy.

[36] Kazakov M., Klimov O., Dik P., Shaverina A., Pereyma V.Y., Noskov A. (2017). Hydroconversion of oil shale on natural mineral matrices. Pet. Chem..

[37] Heydari M., Rahman M., Gupta R. (2015). Kinetic study and thermal decomposition behavior of lignite coal. Int. J. Chem. Eng..

[38] Johns W.D. (1979). Clay mineral catalysis and petroleum generation. AREPS.

[39] Brown D., Rhodes C. (1997). Brønsted and Lewis acid catalysis with ion-exchanged clays. Catal. Lett..

[40] Bruce C.H. (1984). Smectite dehydration—Its relation to structural development and hydrocarbon accumulation in northern Gulf of Mexico basin. AAPG Bull..

[41] Yan L., Stucki J.W. (1999). Effects of Structural Fe Oxidati.on State on the Coupling of Interlayer Water and Structural Si− O Stretching Vibrations in Montmorillonite. Langmuir.

[42] (2016). Petroleum Geological Exploration Professional Standardization Committee. Ananlysis Mothed for Family Composition of Rock Extracts and Crude Oil.

[43] (2010). National Petroleum Products and Lubricants Standardization Technical Committee. Test Method for Separation of Asphalt into Four Fractions.

[44] (2012). Determination of Thermal Property Parameters of the Wax Precipitation in Crude Oil. Test Method by Differential Scanning Calorimetry.

